# Biophysical modeling of excitation/inhibition imbalance in Alzheimer’s disease

**DOI:** 10.64898/2026.09.30.755800

**Published:** 2026-10-07

**Authors:** Qin Deng, Cici Chen, Basilis Zikopoulos, Arash Yazdanbakhsh

## Abstract

**INTRODUCTION:**

Alzheimer’s disease (AD) involves early sensory and perceptual changes that can precede memory decline. We developed a two-layer rate-based neural model of early and higher visual areas with feedforward, feedback, and recurrent connections to examine how excitatory/inhibitory (E/I) balance shapes visual motion perception in AD.

**METHODS:**

Using Motion-Induced Position Shift and Altered Motion Repulsion stimuli, we factorially manipulated excitatory (E) and inhibitory (I) subfield power and connectivity to examine their effects on illusion representation across visual layers.

**RESULTS:**

Increased E, decreased I, and increased E/I reduced illusion representation, consistent with reduced illusion susceptibility in AD, whereas the converse increased illusion representation, reflecting neurotypical processing. Higher-level layer 2 representations were more resistant to E/I perturbations than lower-level layer 1. Illusion representation was less affected by E/I increases in intra-areal (recurrent) connectivity than in inter-areal (feedforward/feedback) connectivity.

**DISCUSSION:**

This mechanistic framework links E/I balance and connectivity to altered visual motion illusion representations in AD and suggests that E/I and connectivity measures may provide probes of early AD neurobiology. The framework may also generalize beyond vision.

## Full Text Availability

The license terms selected by the author(s) for this preprint version do not permit archiving in PMC. The full text is available from the preprint server.

